# Supplementary material for: Structural basis for DNA unwinding at forked dsDNA by two coordinating Pif1 helicases
Source: Nat Commun. 2019 Nov 26;10:5375. doi: 10.1038/s41467-019-13414-9 (PMC6879534; doi:10.1038/s41467-019-13414-9)
Supplement: Supplementary file 1 — Supplementary Information [file 41467_2019_13414_MOESM1_ESM.pdf]

Supplementary Information

Structural Basis for DNA Unwinding at Forked dsDNA by two coordinating Pif1 helicases

Nannan Su[1,2,#], Alicia K Byrd[3#], Sakshibeedu R Bharath[2,6], Olivia Yang[4,6], Yu Jia[1], Xuhua Tang[2], Taekjip Ha[4*], Kevin D. Raney[3]* and Haiwei Song[1,2,5]*

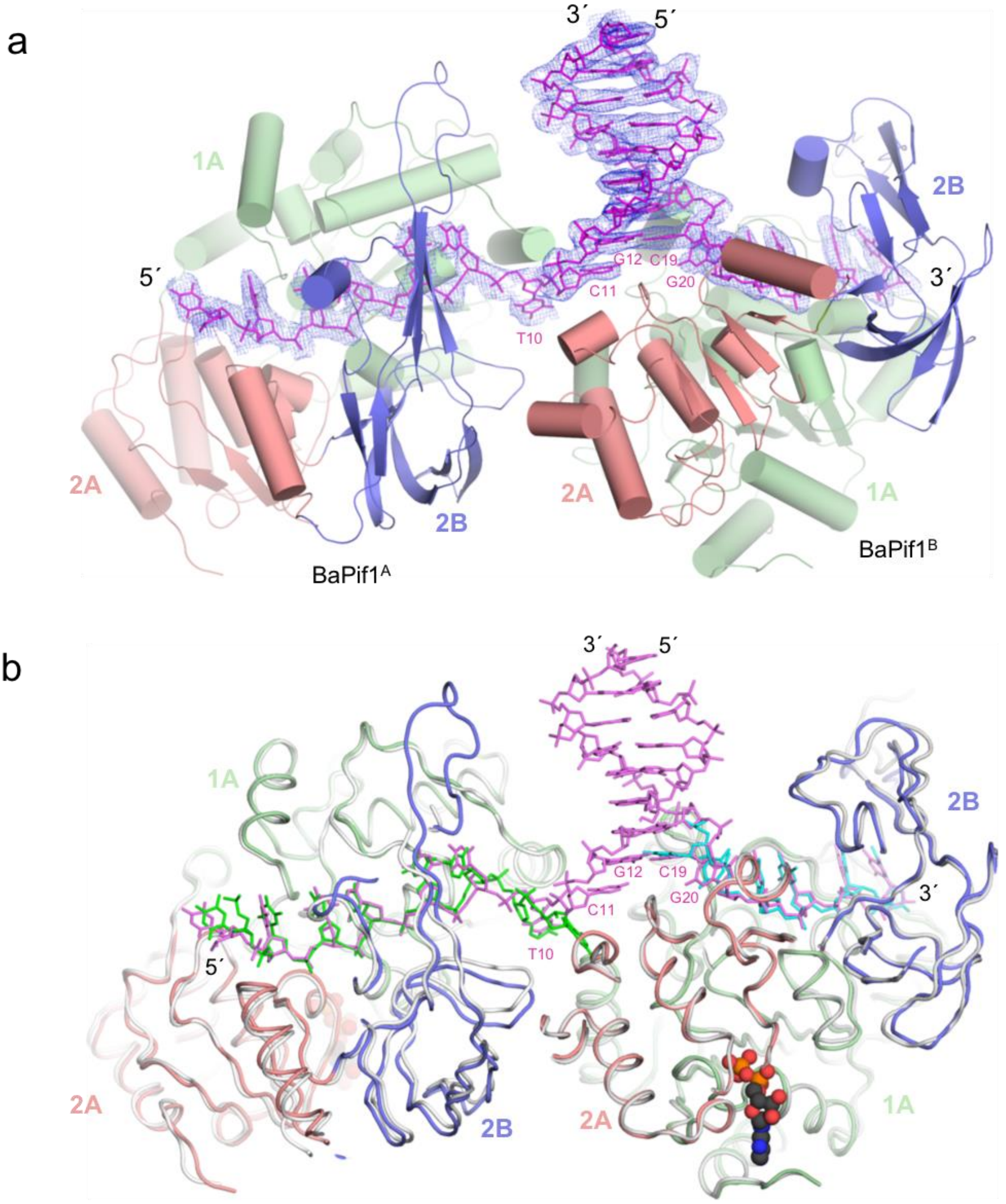

Superposition of BaPif1^A and BaPif1-dH (PDBid: 5FHD, in grey with ssDNA in bright green)

Superposition of BaPif1^B and BaPif1-dT (PDBid: 5FHE, in grey with ssDNA in cyan)

Supplementary Figure 1. Two BaPif1 molecules bound to each fork of the dual forked dsDNA (**a**) BaPif1$_A$ binds only to the 5´ arm of the fork while BaPif1$_B$ binds to the arm and the ss/dsDNA junction. The individual domains 1A, 2A and 2B of BaPif1$_{A\ and\ B}$ are colored distinctly. 2B domain of BaPIf1$_A$ interacts with 2A domain of BaPif1$_B$. 2F$_O$- F$_C$ density contoured at 1σ, observed in the crystal structure around the forked dsDNA is shown as blue mesh with DNA in sticks. (**b**) The conformations of BaPif1$_A$ and BaPif1$_B$ are similar to those observed in BaPif1-dH (PDB id: 5FHD) and BaPif1-dT (PDB id:5FHE).

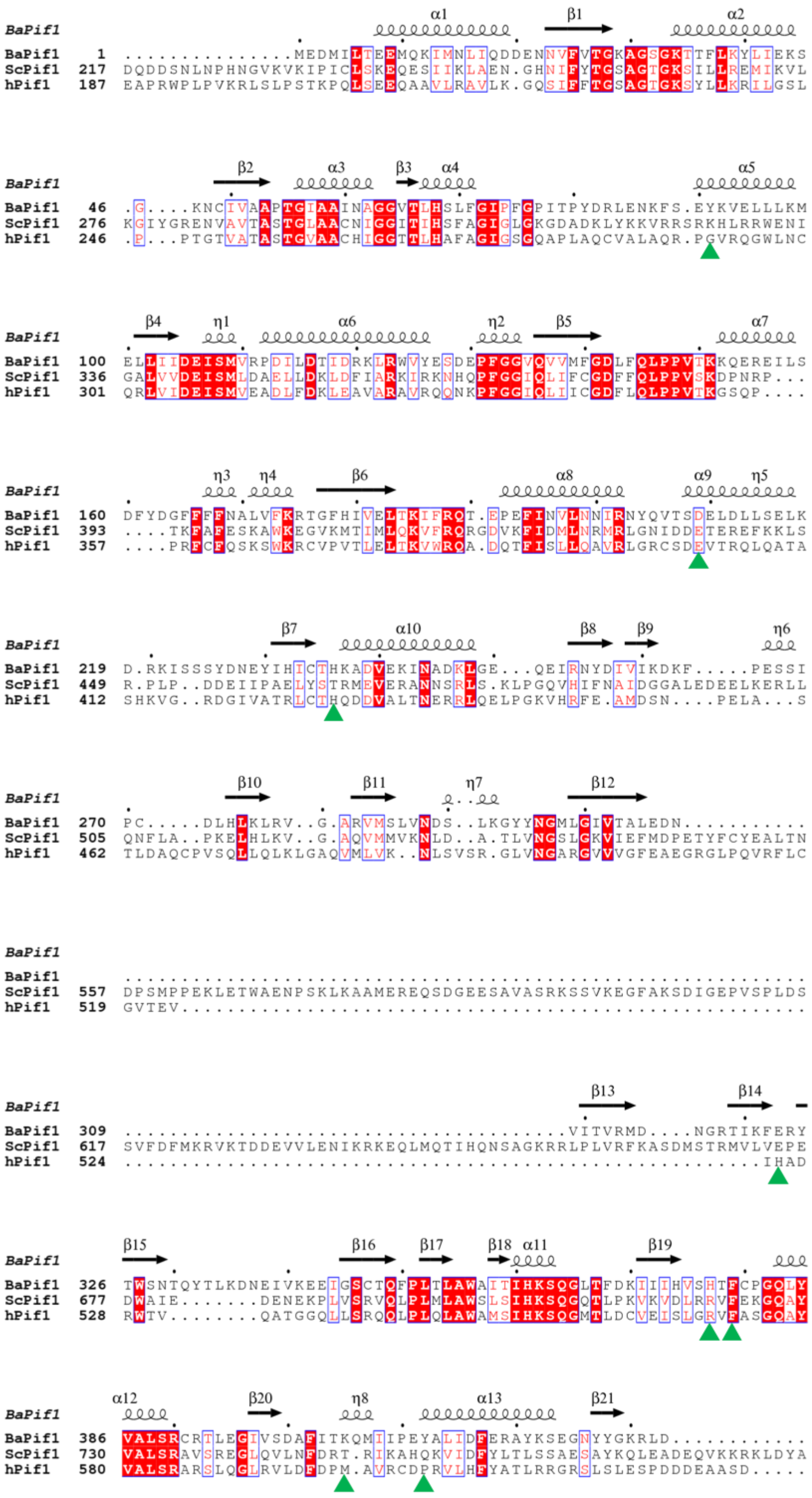

Supplementary Figure 2. Structure based sequence alignment of BaPif1, ScPif1 and hPif1
The sequence alignment is based on the superposition of the crystal structures of ssDNA bound BaPif1 (PDB id: 5FHD) and ScPif1 (PDB id: 5O6B). The secondary structural elements of BaPif1 are marked on top of the sequence alignment. Residues mutated in the current study, Tyr91, Asp209, His236, Glu323, His377, Phe379, Lys405, and Tyr412 are marked with green triangles. Phe379 and Glu323 of BaPif1 are structurally equivalent to Phe723 and Glu674 of ScPif1, respectively.

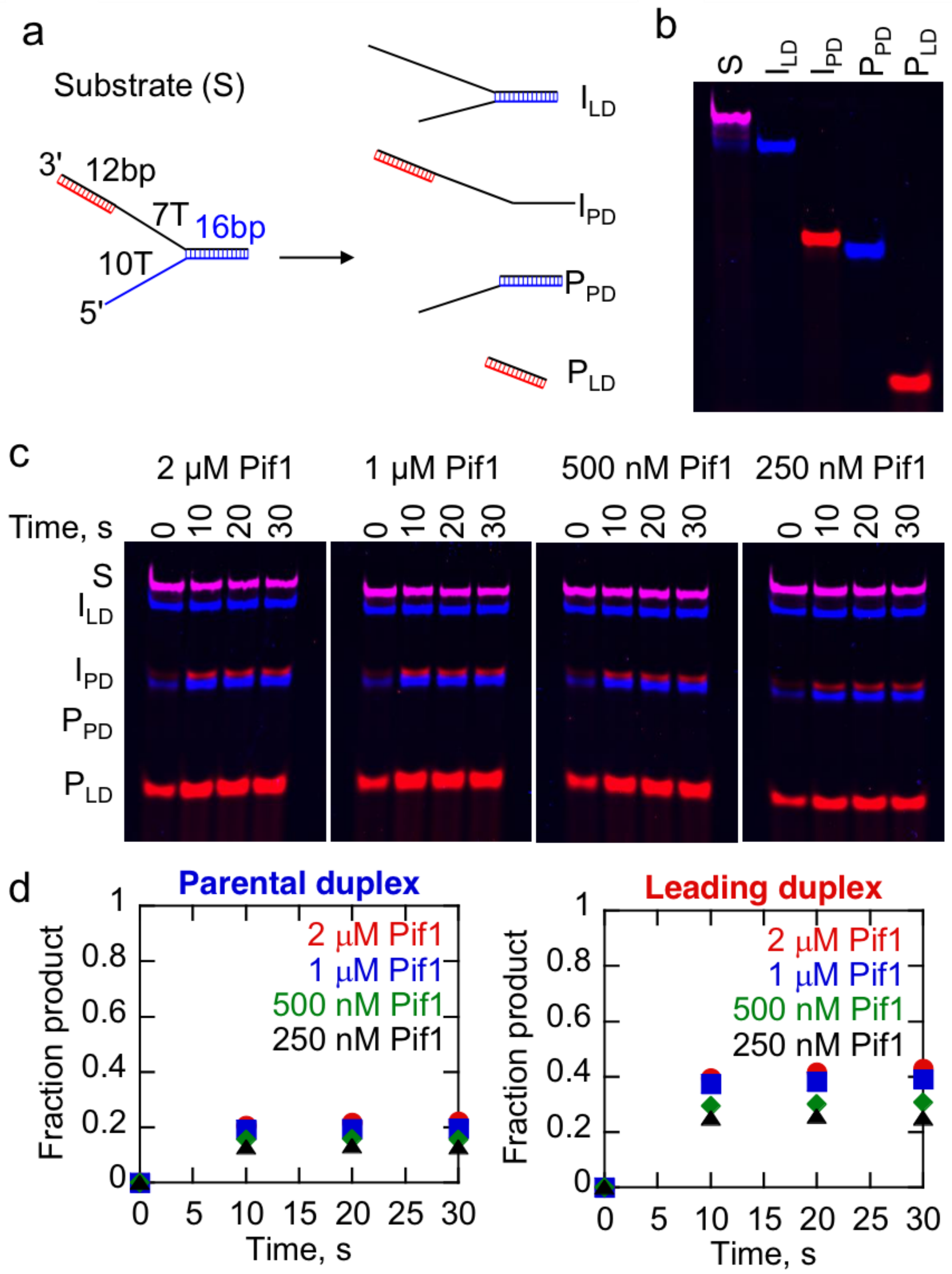

Supplementary Figure 3. Single turnover unwinding of a dual duplex substrate by BaPif1 (**a**) The substrate contains a fluorescein labeled forked duplex (blue) called the parental duplex and a Cy5 labeled duplex on the 3´ arm of the fork (red) termed the leading duplex. (**b**) The substrate (S), intermediates (I_{LD} with the leading duplex unwound and I_{PD} with the parental duplex unwound) and products (P_{PD} is the trapped product of parental duplex unwinding and P_{LD} is the trapped product of leading duplex unwinding) can be separated by native PAGE. (**c**) Unwinding assay with decreasing concentrations of BaPif1 for a substrate containing a 16 bp parental duplex (blue) and a 12 bp leading duplex (red). (**d**) The quantity of product formed on both strands increases as the concentration of BaPif1 increases up to 1 μM and the product formation is saturated at 1 μM. Source gels and quantification data are provided as a Source Data file.

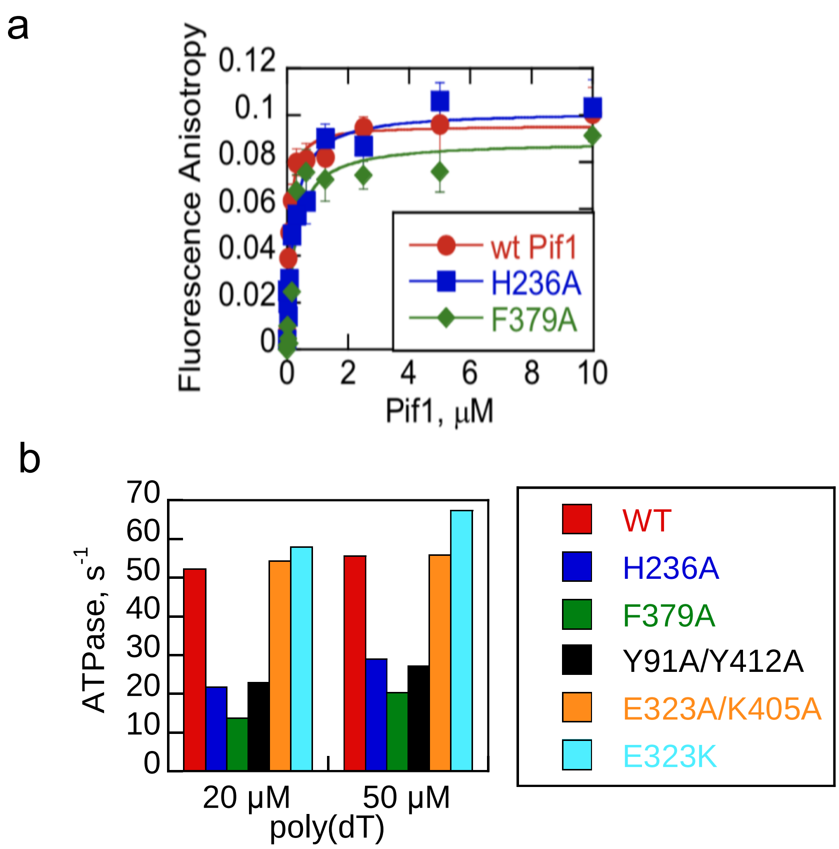

Supplementary Figure 4. Measurements of ssDNA Binding affinity and ATPase activity of wtBaPif1 and its variants (a) wtBaPif1 binds ssDNA with a K_d of 74 ± 9 nM. H236A BaPif1 binds ssDNA with a K_d of 200 ± 50 nM. F379A BaPif1 binds ssDNA with a K_d of 250 ± 80 nM. n=3 independent experiments. (**b**) The ATPase activity of wtBaPif1 and its variants, H236A, F379A, Y91A/Y412A, E323A/K405A, and E323K was measured on poly-dT. n=2 independent experiments. Source quantification data are provided as a Source Data file.

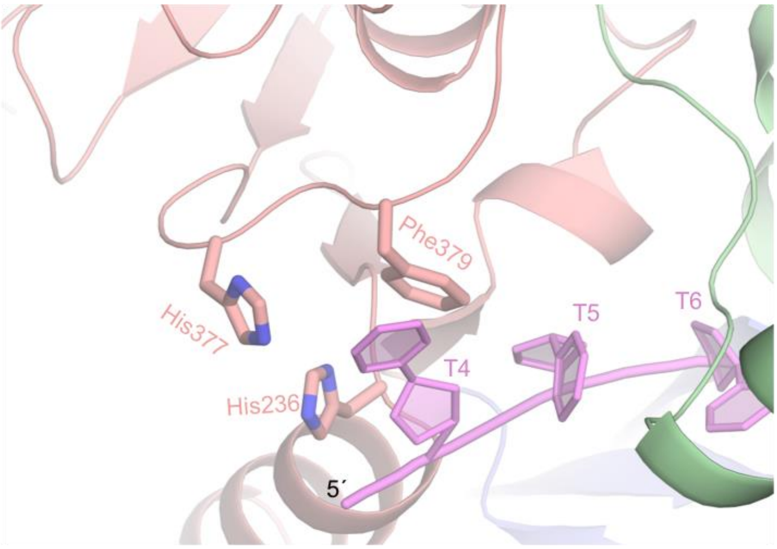

BaPif1^{A} bound to 5´ arm of forked dsDNA

Supplementary Figure 5. Interactions of Phe379, His236 and His377 of BaPif1$_{A}$ with the 5´ terminal nucleotide of the forked-dsDNA

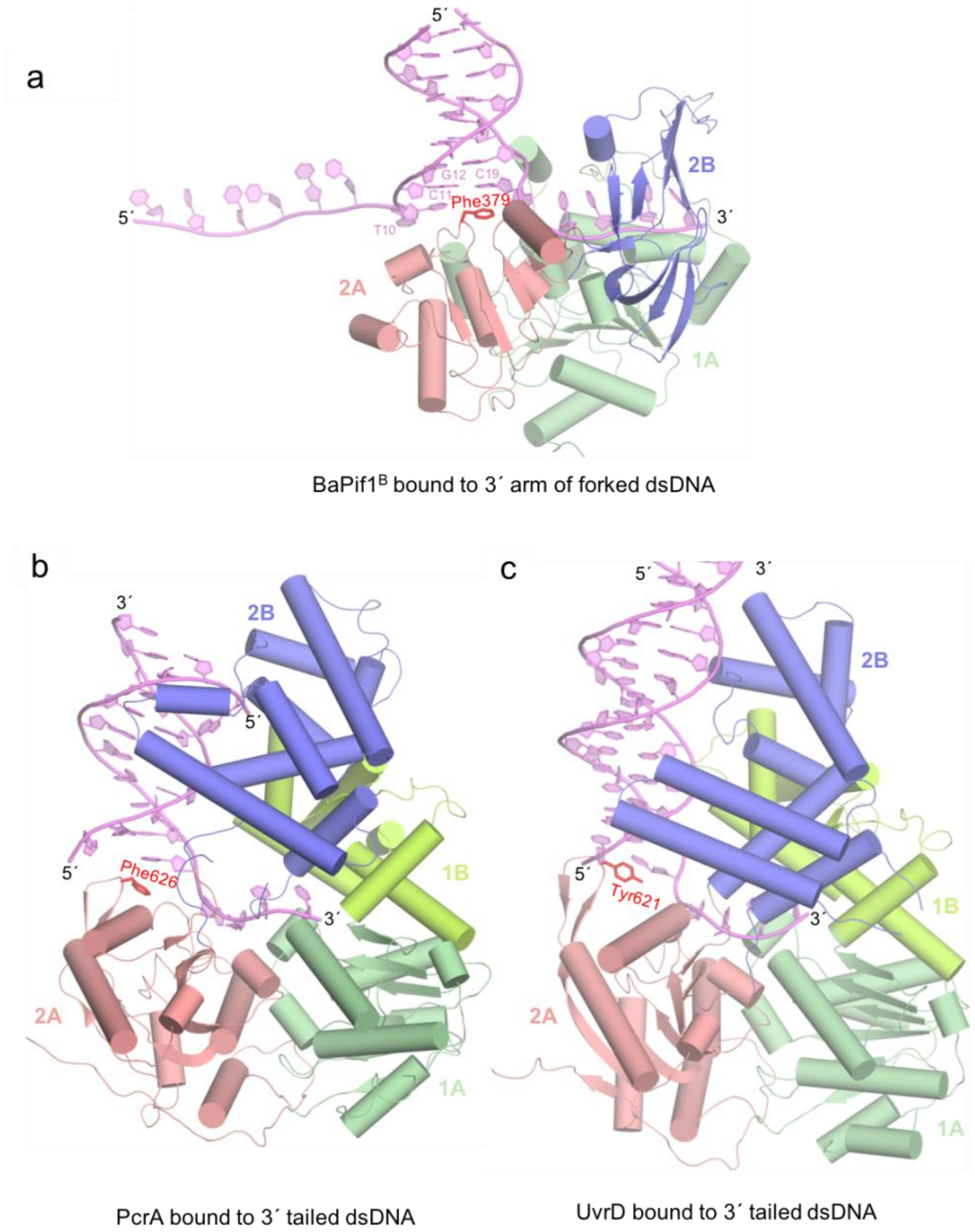

Supplementary Figure 6. Comparison of BaPif1$_B$ bound at 3´ ss/dsDNA junction with PcrA and UvrD bound to the 3´ tailed dsDNA (**a**) BaPif1 shares similar domains with other SF1 helicases namely, 1A and 2A colored in pale green and salmon. BaPif1 has a SH3 accessory 2B domain similar to other SF1B helicases shown in blue. BaPif1$_B$ does not translocate in the 3´ to 5´ direction unlike PcrA and UvrD. (**b**) PcrA and (**c**) UvrD have helical accessory domains 1B and 2B (shown in green and blue) in addition to the conserved domains 1A (pale green) and 2A (salmon). PcrA and UvrD share a "pin" after motif VI and appear to peel away the 3´ tail off the duplex at a right angle, by positioning the pin at the ss/dsDNA junction. BaPif1 has a wedge which serves a similar function between motifs Ia and II; however, since BaPif1 translocates in the opposite direction of PcrA and UvrD, the wedge is located on the opposite side of the protein and is not positioned for unwinding the duplex from the 3′ tail.

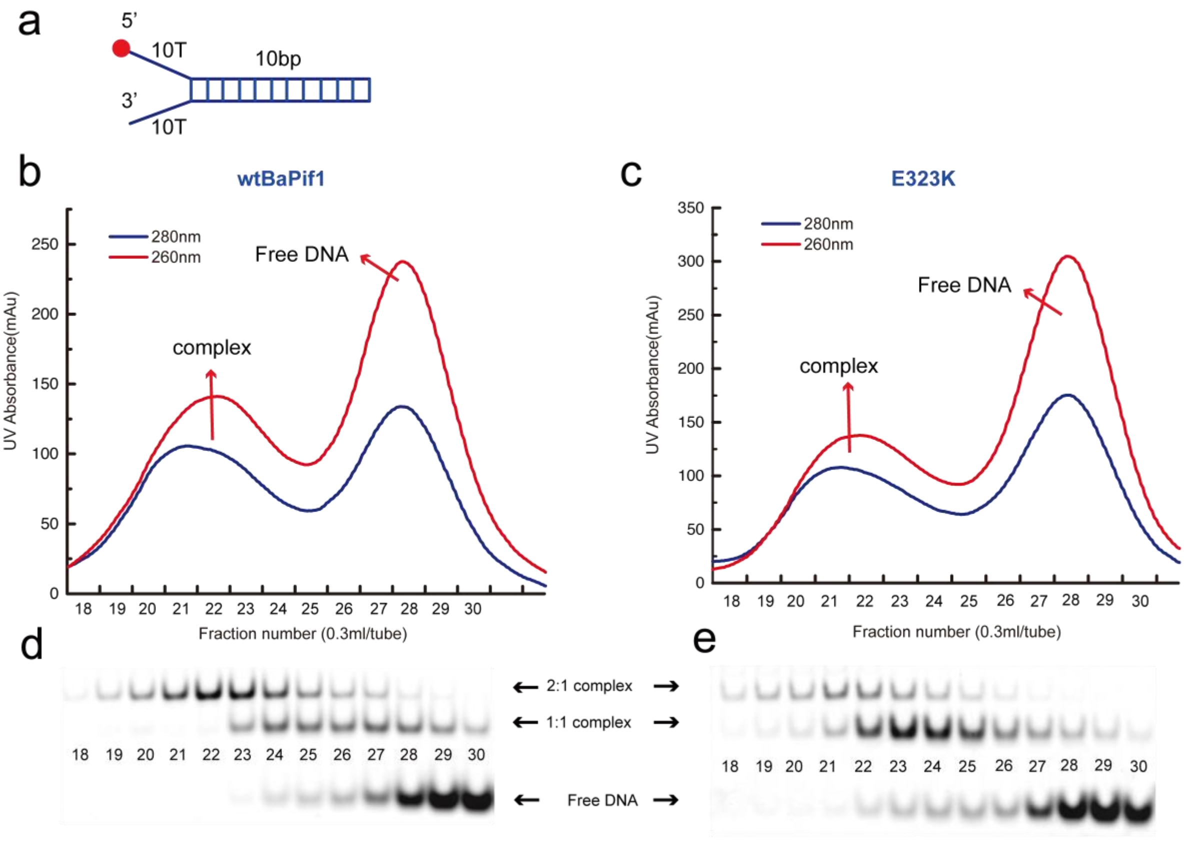

Supplementary Figure 7. Dimerization on DNA is weakened for the E323K variant
(**a**) Single forked DNA substrate labeled with FAM at 5´ tail of the fork. Gel filtration profiles of wtBaPif1 (**b**) and its E323K variant (**c**) complexed with forked DNA at 2:1 molar ratio in the presence of ADP AlF4-. The samples from fractions 18-30 were run on native PAGE (**d** and **e**) to visualize the formation of 2:1 and 1:1 protein-DNA complexes. Source native PAGE gels are provided as a Source Data file.

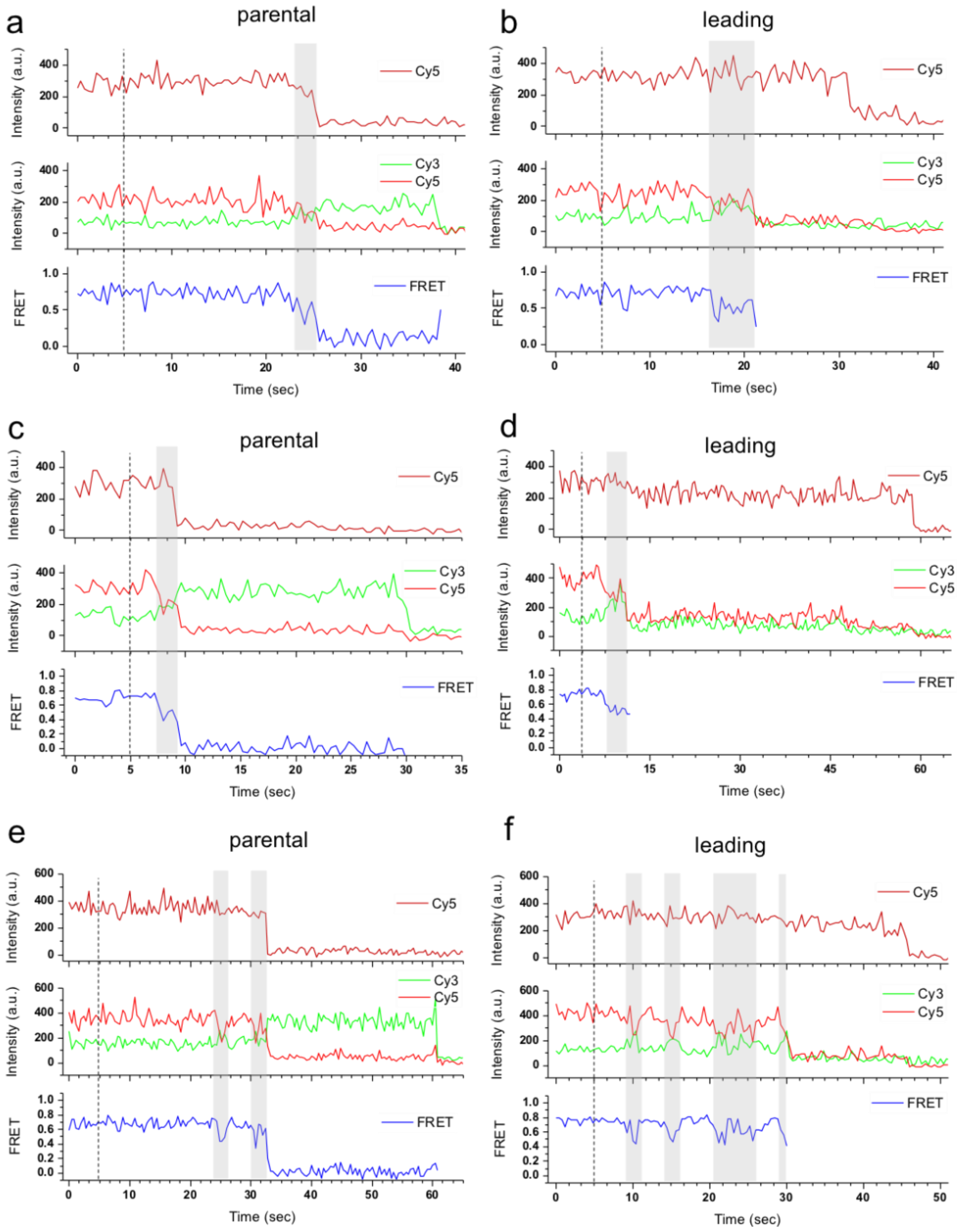

Supplementary Figure 8. smFRET traces for unwinding a dual duplex substrate by wtBaPif1 and its variants Mid-FRET intermediate states (gray) are observed with Cy3 excitation (middle), shortly followed by loss of signal of Cy3. Unwinding of the leading duplex (loss of signal) was observed with direct Cy5 excitation (top). The enzyme (wtBaPif1 or E323A/K405A or Y91A/Y412A) and ATP were flown in at 5 s (dashed line). Traces (**a**) and (**b**) correspond to those obtained with wtBaPif1; traces (**c**) and (**d**) with E323A/K405A; and traces (**e**) and (**f**) with Y91A/Y412A. (**a**), (**c**), and (**e**) represent example smFRET trace depicting unwinding of parental duplex before leading duplex. (**b**), (**d**), and (**f**) represent example smFRET trace depicting unwinding of leading duplex before parental duplex.

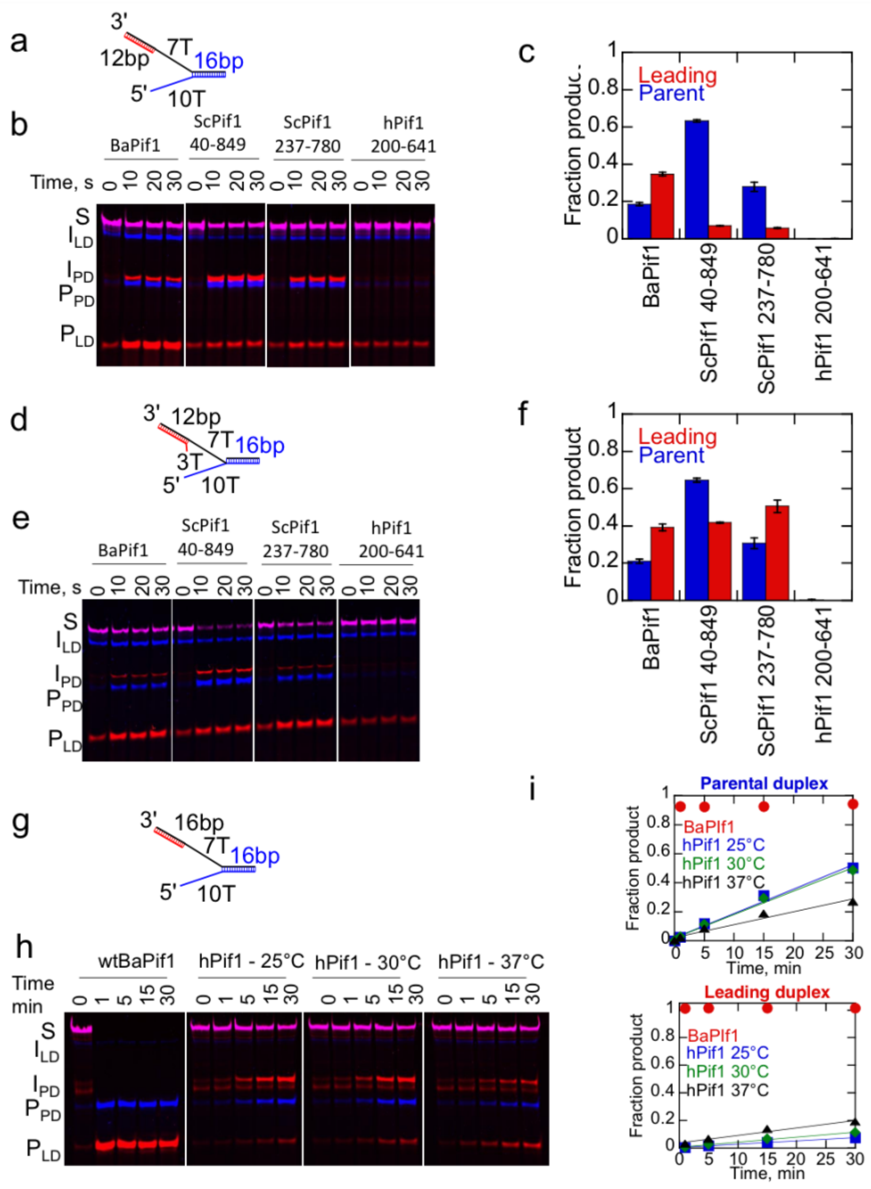

Supplementary Figure 9. Unwinding by BaPif1, ScPif1(40-859), ScPif1(238-780) and hPif1(200-641) (**a**) Single turnover unwinding of substrates with a 16 bp parental duplex (blue) and 12 bp leading duplex (red) by BaPif1, nuclear ScPif1 (40-849), ScPif1 helicase domain (238-780), and hPif1 revealed differences in the quantity of each duplex unwound (**b**). (**c**) Quantification of the products of single turn over unwinding reactions with BaPif1, ScPif1(40-859), ScPif1(238-780) and hPif1(200-641). (**d**) Single turnover unwinding of substrates with a 16 bp parental duplex (blue) and 12 bp leading duplex with a 3T overhang (red) by BaPif1, nuclear ScPif1 (40-849), ScPif1 helicase domain (238-780), and hPif1 revealed BaPif1 and ScPif1 have similar activities for unwinding forked duplexes (**e**). (**f**) Quantification of the products of single turn over unwinding of forked duplexes with BaPif1, ScPif1(40-859), ScPif1(238-780) and hPif1(200-641). (**g**) Multi-turnover experiments demonstrate that hPif1(200-641) unwinds both duplexes (**h**). (**i**) Quantification of the products of multiple turnover unwinding reactions with hPif1(200-641) at 25 °C, 30 °C and 37 °C. Source gels and quantification data are provided as a Source Data file.

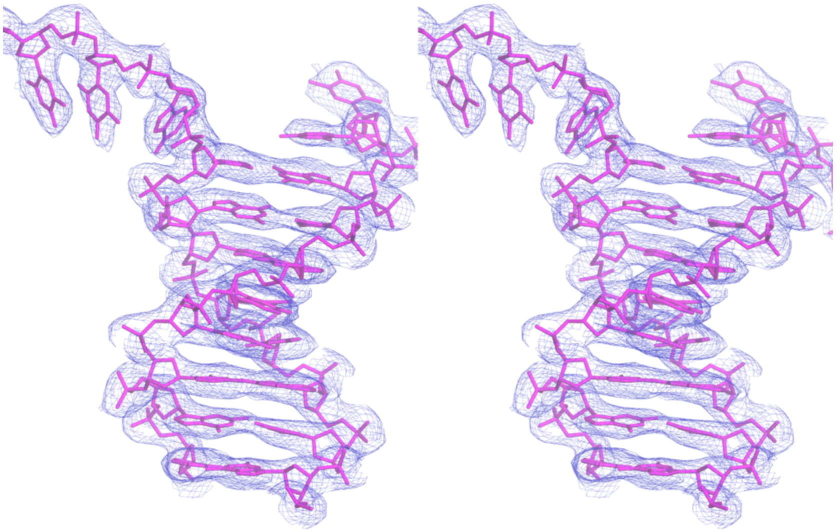

Supplementary Figure 10. Stereo image of a portion of the electron density map. $2F_o - F_c$ density map contoured at 1σ around the forked dsDNA is shown as blue mesh with DNA in sticks.

Supplementary Table 1. Oligonucleotide sequences for unwinding experiments

Substrate	Sequence
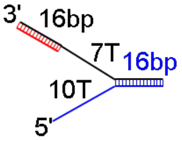	/56-FAM/FTTTTTTTTTTCGCTGATGTCGCCTGG<br>/5Cy5/CCTGTCTGGTCACTGC<br>CCAGGCGACATCAGCGTTTTTTTGCAGTGACCAGACAGG
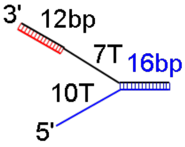	/56-FAM/FTTTTTTTTTTCGCTGATGTCGCCTGG<br>/5Cy5/CCGTGAGTACGC<br>CCAGGCGACATCAGCGTTTTTTTTTTGCGTACTCACGG
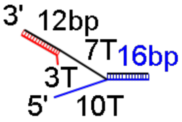	/56-FAM/FTTTTTTTTTTCGCTGATGTCGCCTGG<br>/5Cy5/CCGTGAGTACGCTTT<br>CCAGGCGACATCAGCGTTTTTTTTTTGCGTACTCACGG

Supplementary Methods

DNA Binding

Fluorescein labeled DNA (2.5 nM T_{15}-3′F) was incubated with varying concentrations of BaPif1 (wildtype or variants) in 20 mM Tris, pH 7.5, 50 mM NaCl, 2 mM DTT, 0.1 mg·ml^{-1} BSA, 5% glycerol for 30 min in the dark. A PerkinElmer Life Sciences 1420 Victor^{3}V plate reader was used to measure polarization with excitation at 485 nm and emission at 535 nm. The dissociation constant (K_d) was determined by fitting the data to a hyperbola. Data is the average and standard deviation of three independent experiments.

ATP hydrolysis

A spectrophotometric assay in which the hydrolysis of ATP is coupled by pyruvate kinase and lactate dehydrogenase to NADH oxidation was used to measure ATP hydrolysis. 50 nM BaPif1 (wildtype or variant) was added to 10 mM Tris, pH 7.5, 50 mM NaCl, 0.1 mM EDTA, 2 mM DTT, 0.1 mg·ml^{-1} BSA, 5 mM ATP, 10 mM $MgCl_2$, 4 mM phosphoenolpyruvate, and 15 units·ml^{-1} pyruvate kinase/lactate dehydrogenase, 5% glycerol. The change in absorbance at 380 nm was monitored upon addition of poly(dT). The rate of ATP hydrolysis, which correlates directly with the rate of NADH oxidation, was calculated using the extinction coefficient for NADH at 380 nm (1210 L·mol^{-1}·cm^{-1}).
